# Ca^2+^ trapping by allosteric coupling explains species differences in TRPM2 inactivation reversibility

**DOI:** 10.1038/s42003-026-10197-w

**Published:** 2026-05-07

**Authors:** Adam Bartok, László Csanády

**Affiliations:** 1 Department of Biochemistry, Semmelweis University, Budapest, Hungary; 2 HUN-REN-SE Ion Channel Research Group, Budapest, Hungary; 3 HCEMM-SE Molecular Channelopathies Research Group, Budapest, Hungary

**Keywords:** Molecular conformation, Ion transport, Neurophysiology, Molecular evolution

## Abstract

The Ca^2+^ permeable cation channel TRPM2 is expressed in neurons of the hypothalamic thermoregulatory center, and contributes to body temperature regulation. Its activation gate opens upon binding of cytosolic Ca^2+^ and ADP ribose, but its distinct inactivation gate closes upon prolonged ligand exposure. In contrast to inactivation of most other neuronal channels, inactivation of human TRPM2 (hsTRPM2) is irreversible, but neither the mechanistic reason nor the evolution of that unique feature is known. Here we find that for the zebrafish ortholog (drTRPM2) inactivation is readily reversible upon ligand removal. Using a combination of electrophysiology, kinetic modeling, and thermodynamic analysis we reveal strong allosteric coupling between the extracellular inactivation gate and the cytosolic Ca^2+^ binding site: inactivation gate closure traps Ca^2+^ in its binding site. Moreover, we demonstrate that for hsTRPM2 inactivation is also technically reversible, but hsTRPM2 channels have evolved to bind Ca^2+^ so tightly in the inactivated state, that recovery requires lowering free [Ca^2+^] to subnanomolar levels. These findings delineate the appearance and evolution of TRPM2 inactivation in vertebrates, and explain why the human channel remains trapped in the inactivated state under physiological conditions.

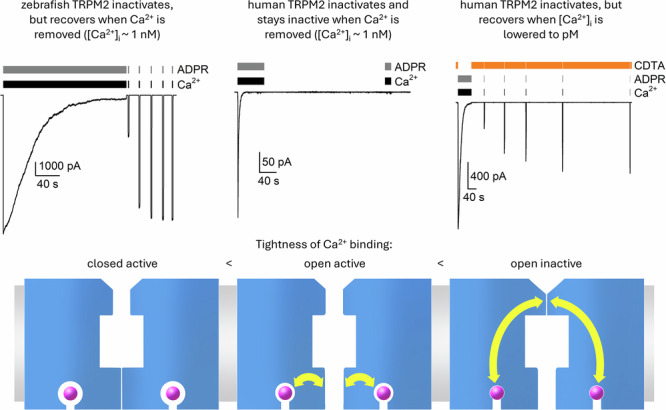

## Abstract (web summary)

A combination of electrophysiology, kinetic modeling, and thermodynamic analysis reveals strong allosteric coupling in TRPM2 channels: closure of the extracellular inactivation gate traps the activating Ca^2+^ ion in its cytosolic binding site.

## Introduction

Transient Receptor Potential melastatin 2 (TRPM2) is a Ca^2+^ permeable cation channel highly expressed in multiple regions of the brain^1–4^, the peripheral sensory nervous system^5–7^, and other tissues including immune cells^2,3^ and pancreatic beta cells^8^. TRPM2 is a highly temperature-sensitive “thermoTRP” channel^9,10^ which contributes to the central regulation of body temperature by the preoptic area (POA) of the hypothalamus^4,11,12^ and to peripheral heat sensation by dorsal root ganglion neurons^5–7^. Under pathological conditions TRPM2 activity contributes to neurodegeneration in Alzheimer’s disease^13,14^, Parkinson’s dementia and amyotrophic lateral sclerosis^15^.

**Fig. 1:**
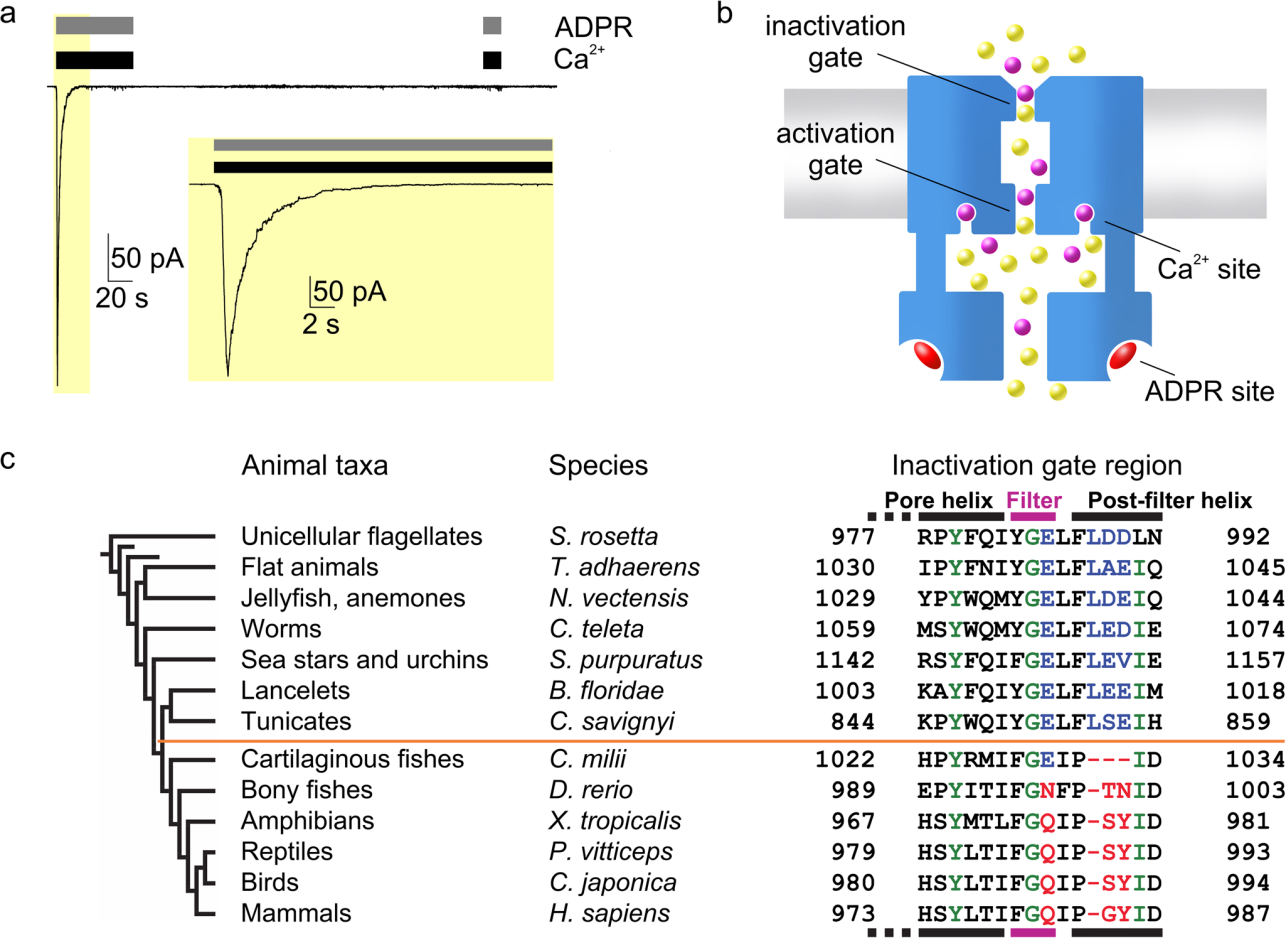
Time course, structural background, and evolution of TRPM2 inactivation. **a** Inside-out patch current from an HEK-293 cell stably expressing hsTRPM2, evoked by cytosolic exposure to saturating (111 μM) Ca^2+^ (*black bar*) + saturating (32 μM) ADPR (*gray bar*). Temperature was 37^o^C, membrane potential was -80 mV, bath solution also contained 10 μM dioctanoyl-PIP_2_ and 200 μM AMP (see Methods). *Inset* shows inactivation time course at an expanded time scale. **b** Cartoon depicting longitudinal section of conserved structural Layers 1-3 of TRPM2 (*marine*). *Purple spheres*, Ca^2+^ ions; *yellow spheres*, Na^+^ ions; *red ovals*, ADPR; *gray rectangles*, membrane. **c** Sequence alignment of the pore region (pore helix, selectivity filter and post-filter helix) for TRPM2 orthologs chosen to span the entire evolutionary distance from choanoflagellates to man. *Horizontal orange line* demarks invertebrates from vertebrates. Note replacement of *blue triplet* in invertebrates by *red doublet* in vertebrates.

The TRPM2 channel is activated by simultaneous binding of cytosolic ADP ribose (ADPR) and Ca^2+^ ^2,16–18^. The response of the human channel (hsTRPM2) to these agonists is shaped by two processes, activation and inactivation (Fig. 1a). Inactivation of hsTRPM2, observed in inside-out patches upon prolonged agonist exposure, is irreversible: channel activity cannot be recovered even after several minutes in an agonist-free solution (Fig. 1a, *right*). Although TRPM2 also requires membrane phosphatidylinositol-4,5-bisphosphate (PIP_2_) for activity^19^, inactivation does not reflect membrane PIP_2_ depletion, as it cannot be prevented or reversed by superfusion with exogenous PIP_2_^18^. For most other neuronal voltage-gated (e.g., K_V_, Na_V_, Ca_V_) or ligand-gated (glutamate, acetylcholine, GABA, or glycine receptor) channels inactivation/desensitization is fully reversible, allowing channels to become repeatedly activated over prolonged periods of time. Thus, irreversibility of hsTRPM2 inactivation is highly unusual, and neither its physiological rationale nor its mechanistic reason is known.

TRPM2 is a homotetramer, and its four subunits assemble into a four-layered structure^20–22^. The transmembrane (TM) domain (Layer 1) is formed by the six TM helices (S1–S6) of each subunit and – although TRPM2 gating is not voltage sensitive – resembles that of voltage-gated cation channels, consisting of a central pore domain (S5-S6) surrounded by four voltage sensor-like domains (S1-S4). The binding sites for activating Ca^2+^ ions are formed by the cytosolic ends of the S2-S3 helices and a short S2-S3 linker helix^20,21,23^, at a distance of only ~3 nm from the cytosolic pore entrance (Fig. 1b), strategically located to bind Ca^2+^ ions (Fig. 1b, *purple spheres*) entering through the pore^18,23^. Three additional cytosolic layers are built from intertwining long N- and C-terminal polypeptide segments. Layer 2 surrounds a large cytosolic cavity, while Layer 3 accommodates the binding sites for activating ADPR (Fig. 1b, *red ovals*) formed by N-terminal peptide segments (“N-site”). The architecture of structural Layers 1-3 (Fig. 1a) has remained conserved in all TRPM2 orthologs from choanoflagellates to man^20–24^. In contrast, the four C-terminal NUDT9-H domains, which also bind ADPR (“C-site”), assemble into a Layer 4 that is both structurally^20–24^ and functionally^25,26^ variable.

Access of cations to the TRPM2 pore is regulated by two distinct gates. The cytosolic “activation gate” (Fig. 1b), opened by agonist binding, consists of a four-helix bundle formed by the C-terminal ends of S6^20^, and is homologous to the activation gate of most other voltage-gated cation channels^27^. In contrast, mutagenesis studies have pinpointed the “inactivation gate” (Fig. 1b) to the extracellular selectivity filter^19^, suggesting that TRPM2 inactivation resembles slow (C-type) inactivation of Na_V_ and K_V_ channels. That assignment is further supported by the simultaneous evolutionary appearance of inactivation and specific changes in pore sequence in vertebrate TRPM2 orthologs^26^: a single-residue deletion and loss of two negatively charged acidic side chains in the short extracellular “post-filter” helix adjacent to the selectivity filter (Fig. 1c, compare *blue* and *red triplets* above and below *orange line*).

While studying the biophysical properties of the zebrafish (*Danio rerio*) TRPM2 (drTRPM2) channel^28^, an early vertebrate ortholog that already shows inactivation, here we discover that inactivation is reversible for drTRPM2. We thus exploit that model system to address the molecular mechanisms of TRPM2 inactivation and recovery. Our studies reveal strong allosteric crosstalk between the activation gate, the inactivation gate, and the Ca^2+^ binding site, demonstrate that these mechanisms also apply to hsTRPM2, and provide a mechanistic explanation for the irreversibility of hsTRPM2 inactivation under physiological conditions.

## Results

### Inactivated drTRPM2 channels fully recover upon removal of cytosolic ADPR and Ca^2+^

**Fig. 2:**
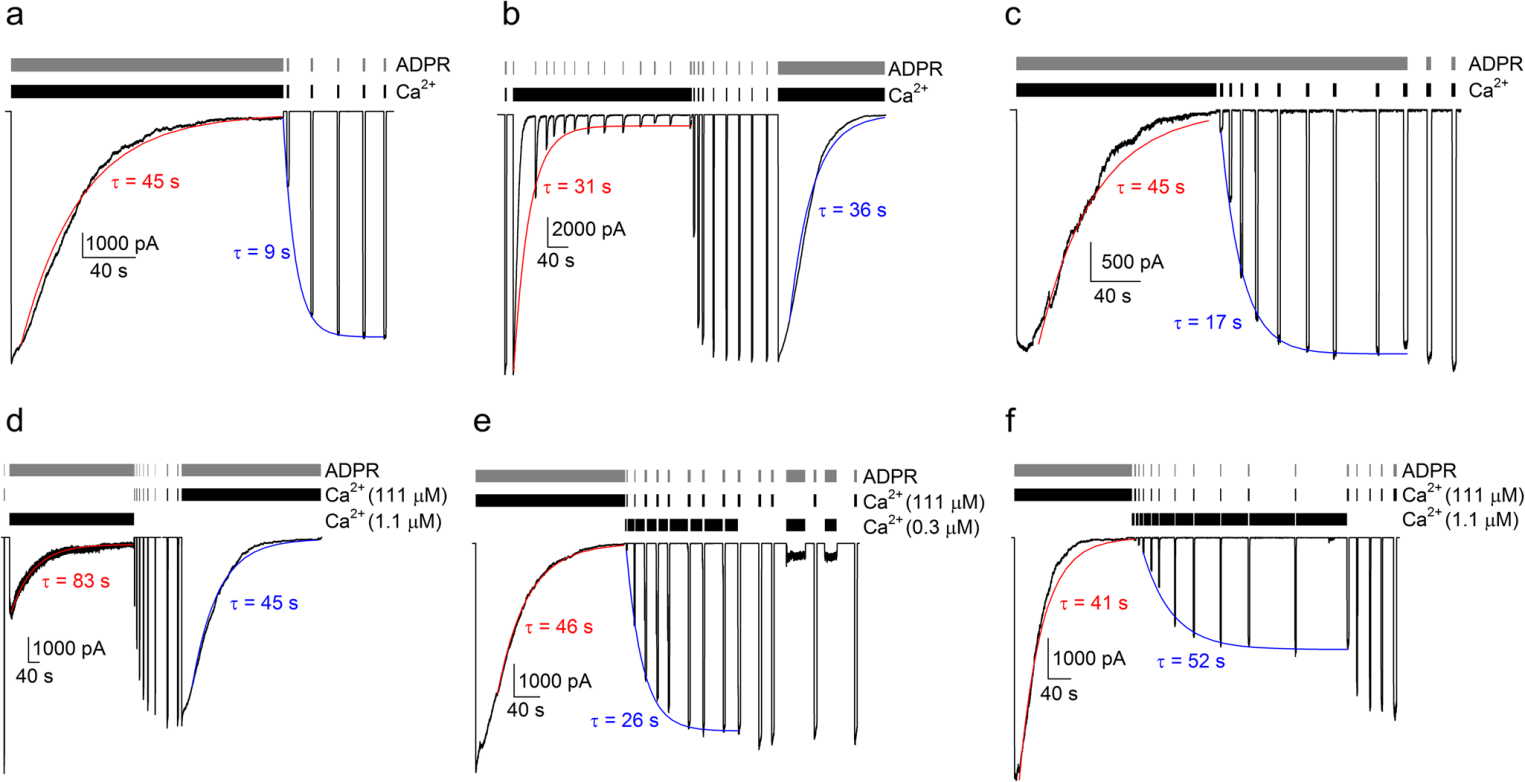
Modulation of drTRPM2 inactivation and recovery by Ca^2+^ and ADPR. **a**–**f** Inside-out patch currents from HEK-293T cells transiently expressing drTRPM2, under experimental conditions as in Fig. 1a, evoked by cytosolic exposure to Ca^2+^ (*black bars*) and ADPR (*gray bars*). In (*a*-*c*) both agonists were applied at saturating concentrations (111 μM Ca^2+^, 32 μM ADPR). In (**d**–**f**), ADPR was saturating (32 μM), while alternating concentrations of Ca^2+^ are marked on the right. In the “zero Ca^2+^” solution (**a**–**f**, periods between *black bars*) free [Ca^2+^] was 1 nM. Time courses of inactivation in (**a**, **c**–**f**) and of the second inactivation in (**b**) were fitted by single exponentials (red and blue curves, time constants plotted). For the time courses of recovery in (**a**, **c**, **e**, **f**), and of the first inactivation in (**b**), envelope curves (*red* and *blue curves*, time constants plotted) were obtained by fitting single exponentials to the maximum points of the current responses to the brief agonist exposures.

To study the biophysical properties of drTRPM2, channel currents were recorded from inside-out patches excised from HEK-293T cells (Fig. 2a-f). The cytosolic membrane surface was continuously superfused with temperature-controlled bath solution, and extracellular (pipette) [Ca^2+^] was kept at ~1 nM to secure perfect control over local [Ca^2+^] around the activating sites. Similarly to hsTRPM2, drTRPM2 channel opening requires simultaneous exposure to ADPR and Ca^2+^, and in the sustained presence of saturating concentrations of both agonists the channel inactivates (Fig. 2a, *left*), albeit slower than the human ortholog (cf., Fig. 1a^26^). However, in stark contrast to hsTRPM2 (Fig. 1a), we found that drTRPM2 currents can be reactivated by ADPR+Ca^2+^ following a period of exposure to ligand-free solution. Under such ligand-free conditions the active channel pool fully recovered, and the recovery time course could be monitored by periodic brief (1-2 s) exposures to saturating ADPR+Ca^2+^ (Fig. 2a, *right*). That unexpected finding identified the zebrafish ortholog as an excellent model system for studying the biophysical mechanisms of TRPM2 inactivation and recovery.

Inactivation and recovery rates of drTRPM2 were insensitive to membrane voltage (Supplementary Fig. 1a), but showed substantial temperature dependence (cf. ^28^,), being slowed by ~3-fold and ~7.5-fold, respectively, upon cooling the patches from 37 °C to 25 °C (Supplementary Fig. 1c, d). All further studies were carried out at a membrane potential of -80 mV and a temperature of 37 °C.

### The active-inactive equilibrium is strongly modulated by Ca^2+^, but little by ADPR binding

The above observations (Fig. 2a) indicate that the active-inactive equilibrium is completely shifted towards inactive for drTRPM2 channels bound to both Ca^2+^ and ADPR, but completely shifted towards active for unliganded channels. We next addressed which of the two ligands affects the equilibrium.

**Fig. 3:**
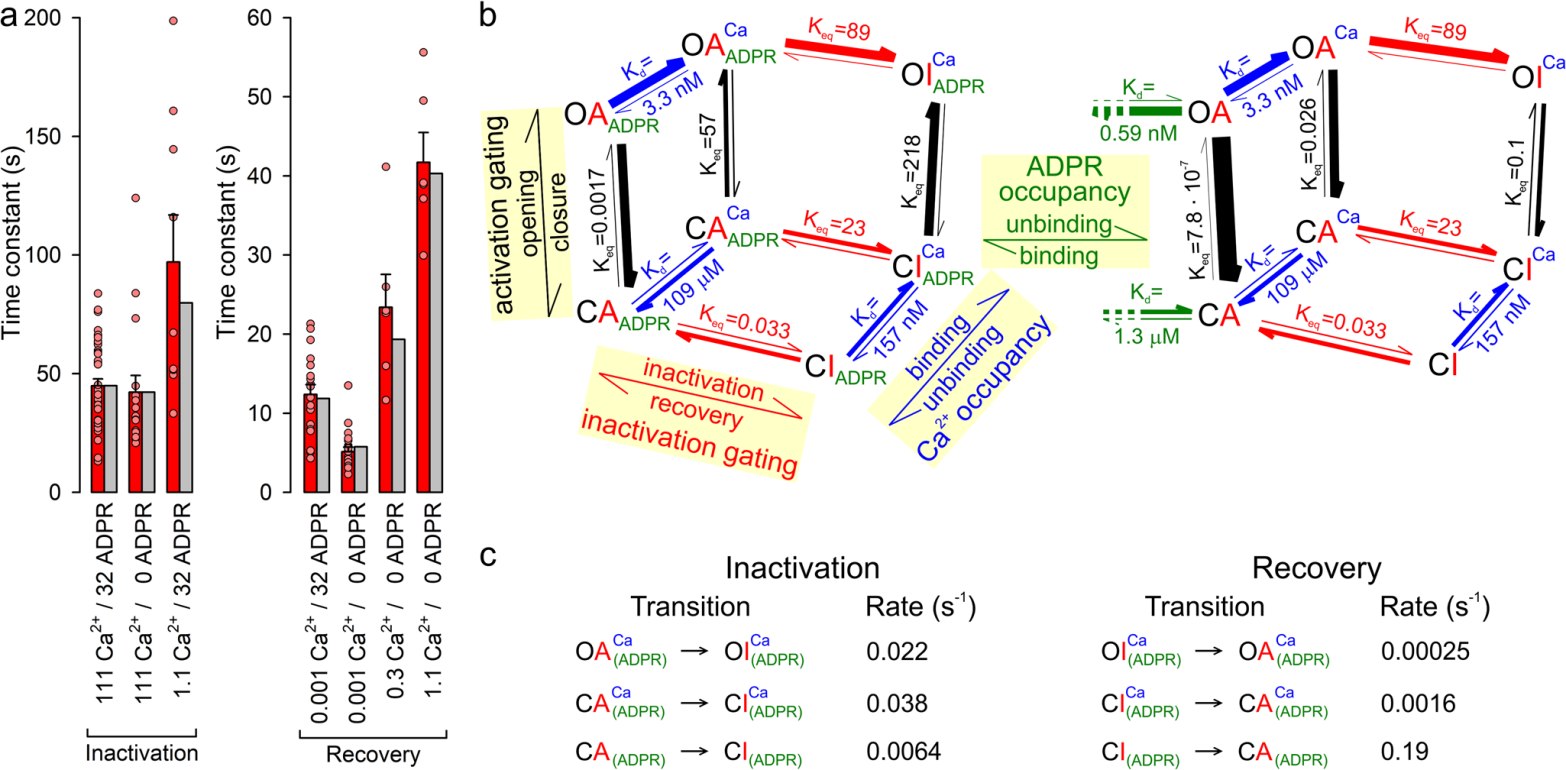
Time constants of inactivation/recovery, and simplified kinetic model. **a** Time constants of inactivation (*left*) and recovery (*right*; calculated using cumulative time spent under recovery conditions) in the presence of the indicated combinations of agonist concentrations, observed experimentally (*red bars*) or predicted (*gray bars*) by the model in (**b**). The experimental data (*red bars*) represent mean ± S.E.M. from n independent patches; n = (from left to right) 39, 16, 9, 18, 19, 6, and 6, respectively. **b** Simplified kinetic model to describe functional states of the activation gate (C or O), the inactivation gate (A or I), and the occupancy of the Ca^2+^ and the ADPR binding site (superscript and subscript, respectively) of drTRPM2, assuming one binding site for each type of ligand. States OI and OI_ADPR_ have small occupancy probabilities and are omitted for clarity. Equilibrium constants (K_eq_) for activation gating (C-O) and inactivation gating (A-I), as well as dissociation constants for both ligands from closed (K_d,c_) and open (K_d,o_) channels are plotted. **c** Absolute rate constants of inactivation (*left*) and recovery (*right*), estimated from the experimentally observed relaxation time constants in (**a**). Compared to the depicted rates of inactivation/recovery, activation gating and ligand occupancies are rapid equilibria; at the time scales of the experiments in Fig. 2a-f the absolute values of the rates for those steps have little impact on the current time courses.

To monitor the process in the presence of Ca^2+^ alone, a saturating concentration of Ca^2+^ was continuously superfused, and the size of the active channel pool was periodically monitored by brief (1-2 s) applications of saturating ADPR (Fig. 2b, *left*). Under such conditions drTRPM2 channels still inactivated, with a time constant similar to that in the presence of ADPR+Ca^2+^ (Fig. 2b, *left* vs. *right*; Fig. 3a, *left*, *1st* vs. *2nd red bar*). However, unlike in the presence of ADPR+Ca^2+^, a small fraction of the channel pool remained active at steady state (compare Fig. 2b, *left* and *right*, see quantitative analysis later). Thus, the active-inactive equilibrium is near-completely shifted towards inactive even for channels bound to just Ca^2+^.

To monitor the process in the presence of ADPR alone, inactivation was first observed in the presence of both ligands, and subsequently Ca^2+^ was removed but ADPR maintained at a saturating concentration (Fig. 2c). Under such conditions the channel pool recovered almost completely, as monitored by periodic brief exposures to Ca^2+^ (Fig. 2c, *right*). Thus, for channels bound to just ADPR the active-inactive equilibrium is near-completely shifted towards active.

These findings indicate that the processes of drTRPM2 inactivation and recovery are primarily modulated by Ca^2+^ binding, and little affected by ADPR binding. Moreover, since in the absence of ADPR the channels inactivate while the activation gate is closed (Fig. 2b, *left*), activation gate opening is not a necessary condition for inactivation to happen.

### Cytosolic [Ca^2+^] modulates both the rate of inactivation and the rate of recovery

The active-inactive equilibrium constant is given by the ratio of the rates of inactivation and recovery. We next addressed which of those two rates is sensitive to [Ca^2+^]. In the presence of ADPR, lowering free [Ca^2+^] from saturating (111 μM) to a near half-saturating level (1.1 μM) slowed inactivation by roughly twofold (Fig. 2d, *left* vs. *right*), prolonging its time constant from 45±3 s (n = 39) to 97±20 s (n = 9) (Fig. 3a, *left*, *1st* vs. *3rd red bar*).

To compare recovery rates under various conditions, recovery time constants were calculated by single-exponential fits to plots of fractional recovery as a function of cumulative time under the recovery condition. Because that time excludes the time intervals of the repeated brief saturating agonist exposures, these “exact” recovery time constants (Fig. 3a, *right*, *red bars*) are substantially shorter than the time constants of the corresponding “envelope” curves (Fig. 2a, c, e, f; *blue curves*), and their inverses provide the actual rate of recovery under a given condition. In the absence of ADPR, raising cytosolic free [Ca^2+^] from ~1 nM (the actual free [Ca^2+^] in our “zero Ca^2+^” bath solution buffered with 1 mM EGTA) to 0.3 μM (Fig. 2e) or 1.1 μM (Fig. 2f) slowed recovery in a [Ca^2+^]-dependent manner (compare *blue fit lines* in Fig. 2a, e, and f; and *2nd* to *4th red bars* in Fig. 3a, *right*).

Thus, Ca^2+^-bound drTRPM2 channels inactivate fast but recover slowly, whereas Ca^2+^-free channels inactivate slowly but recover fast. The Ca^2+^ binding site responsible for this effect must be located on the cytosolic side of the channel, as raising extracellular (pipette) [Ca^2+^] from 1 nM to 0.3 μM affected neither inactivation nor recovery rate (Supplementary Fig. 1b). Moreover, its apparent affinity for Ca^2+^ is comparable to that of the conventional “activating site” (Supplementary Fig. 2a; cf.^28^,), suggesting that Ca^2+^ regulates the activation and the inactivation gate by binding to the same site.

### Kinetic representation of drTRPM2 functional states – a minimal model

Even the simplest functional representation of drTRPM2 – neglecting at a first approximation the homotetrameric nature of the channel – requires consideration of four distinct molecular processes: Ca^2+^ binding/unbinding, ADPR binding/unbinding, activation gate opening/closure, and inactivation gate opening/closure. Thus, a functional state of the channel is defined by a particular combination of those four binary descriptors, yielding 2^4^ = 16 functional states. Denoting the state of the activation gate by C (closed) or O (open), and that of the inactivation gate by A (active) or I (inactive), generates a two-letter representation of the four possible state combinations of the channel gates: CA, OA, CI, and OI. For each of those “gating states” the four possible combinations of ligand occupancies may be denoted by a superscript (for Ca^2+^ occupancy) and a subscript (for ADPR occupancy), e.g., OA, OA_ADPR_, OA^Ca^, ${{\rm{OA}}}^{{{\rm{Ca}}}}_{{\rm{ADPR}}}$. The 16 states can be arranged into two cubes (Fig. 3b), e.g., one for ADPR-bound (*left cube*), and one for ADPR-free (*right cube*) channels. In both cubes of that representation the vertical (*black*) edges depict activation gating, the horizontal *red* edges inactivation gating, and the horizontal *blue* edges Ca^2+^ occupancy. Because the occupancies of Ca^2+^-free OI states are small (see below), those may be omitted for clarity (missing vertices in Fig. 3b). Finally, ADPR binding/unbinding generates connectivities between any two homologous states of the two cubes (Fig. 3b, represented by the two half-dashed horizontal *green double arrows*).

Earlier studies have shown that hsTRPM2 gating is well described by independent binding of ADPR and Ca^2+^^9^, and the current data suggest that ADPR binding little affects inactivation gating (Fig. 2b, c). Thus, the model may be further simplified by assuming that ADPR solely affects activation gating (i.e., vertical equilibria), whereas all the rates along homologous horizontal edges of the two cubes are identical.

### Inactivation gate closure promotes activation gate opening and strengthens Ca^2+^ binding

Each face of the cubes in Fig. 3b represents the equilibria for two distinct molecular events, and its edges form a kinetic loop. If the equilibrium constants (K_eq_) for two parallel edges of a face are largely different, this reflects strong allosteric coupling between the two molecular events represented by that face. Moreover, the four K_eq_ values within a loop are thermodynamically constrained through the principle of detailed balance: for the two alternative pathways that lead from any vertex to the diagonally opposed one the products of the two equilibrium constants involved are identical. Thus, a large ratio between K_eq_ values of two parallel edges must be compensated by an inverse relationship between the K_eq_ values for the complementary two parallel edges: allosteric coupling is necessarily reciprocal. The three faces/loops of the two cubes thus provide three strong mechanistic predictions.

The left vertical faces of both cubes represent allosteric crosstalk between Ca^2+^ occupancy and activation gating, and has been quantitatively described in earlier studies on hsTRPM2^9,18^: Ca^2+^ binding strongly increases the C-O equilibrium constant (compare *black arrows*), in return, the O state binds Ca^2+^ much more tightly (compare *blue arrows*).

The bottom horizontal faces of both cubes represent allosteric coupling between Ca^2+^ occupancy and inactivation gating. In the absence of ADPR, Ca^2+^-bound (closed) drTRPM2 channels inactivate (Fig. 2b, *1st decay*) whereas Ca^2+^-free closed channels recover (Fig. 2a, *right*). I.e., K_eq_ for inactivation is large for Ca^2+^-bound, but small for Ca^2+^-free channels (Fig. 3b, right cube, bottom horizontal face, *red edges*). By thermodynamic necessity that discrepancy must be compensated for by stronger Ca^2+^ binding in the inactivated state (Fig. 3b, right cube, bottom horizontal face, *blue edges*). Moreover, under the assumption that ADPR binding affects only activation gating (Fig. 3b, *vertical transitions*), the same relationships must apply for ADPR-bound channels (left cube, *bottom face*).

Finally, the right vertical faces of both cubes represent allosteric coupling between the conformations of the activation gate and the inactivation gate. Steady-state inactivation of Ca^2+^-bound channels is significantly (p = 0.0025) less complete when the channels are kept closed by the absence of ADPR (Fig. 2b, *1st decay*, I_∞_/I_0_ = 0.045±0.01 (n = 10)), compared to when they are open in the presence of ADPR (Fig. 2b, *2nd decay*, I_∞_/I_0_ = 0.0051±0.0025 (n = 9)). This implies that K_eq_ for inactivation is significantly larger for open than for closed channels (cf., Fig. 3b, both cubes, right vertical face, *red edges*). In return, the closed-open equilibrium of the activation gate must be shifted more towards the open state for inactivated channels (cf., Fig. 3b, both cubes, right vertical face, *black edges*).

Thus, two novel strong qualitative predictions of the model are that inactivation (i) significantly facilitates opening of the activation gate, and (ii) strongly increases the tightness of Ca^2+^ binding.

### Kinetic model quantitates allosteric interaction strengths

Several of the model parameters could be quantitatively estimated from our experimental observations. For inactivation/recovery (Fig. 3b, *red edges*) the time constant (τ) observed under a particular condition (Fig. 3a, *red bars*) reports the sum of the forward and reverse rates (k_inact_+k_recov_ = 1/τ) and fractional steady-state inactivation reports their ratio (k_inact_/k_recov_=K_eq_), allowing estimation of both rates. For activation gate opening (Fig. 3b, *black edges*) the observed open probability (P_o_) in a particular condition allows calculation of the equilibrium constant (K_eq_=P_o_/(1-P_o_)). Finally, dissociation constants of the ligands Ca^2+^ (Fig. 3b, *blue edges*) and ADPR (Fig. 3b, *green edges*) for closed and open channels (K_d,c_, K_d,o_) can be calculated from the midpoints (EC_50_) of macroscopic ligand dose response curves (EC_50_ ~ 1.9 μM for Ca^2+^ and ~ 0.04 μM for ADPR; Supplementary Fig. 2a-b, *red symbols*^28^;) as K_d,o_ = EC_50_·P_o_(unliganded)/P_o_(liganded) and K_d,c_ = EC_50_·(1-P_o_(unliganded))/(1-P_o_(liganded))^29^. Using these estimates as starting values, the parameters were adjusted to satisfy the constraints of detailed balance for each kinetic loop.

**Fig. 4:**
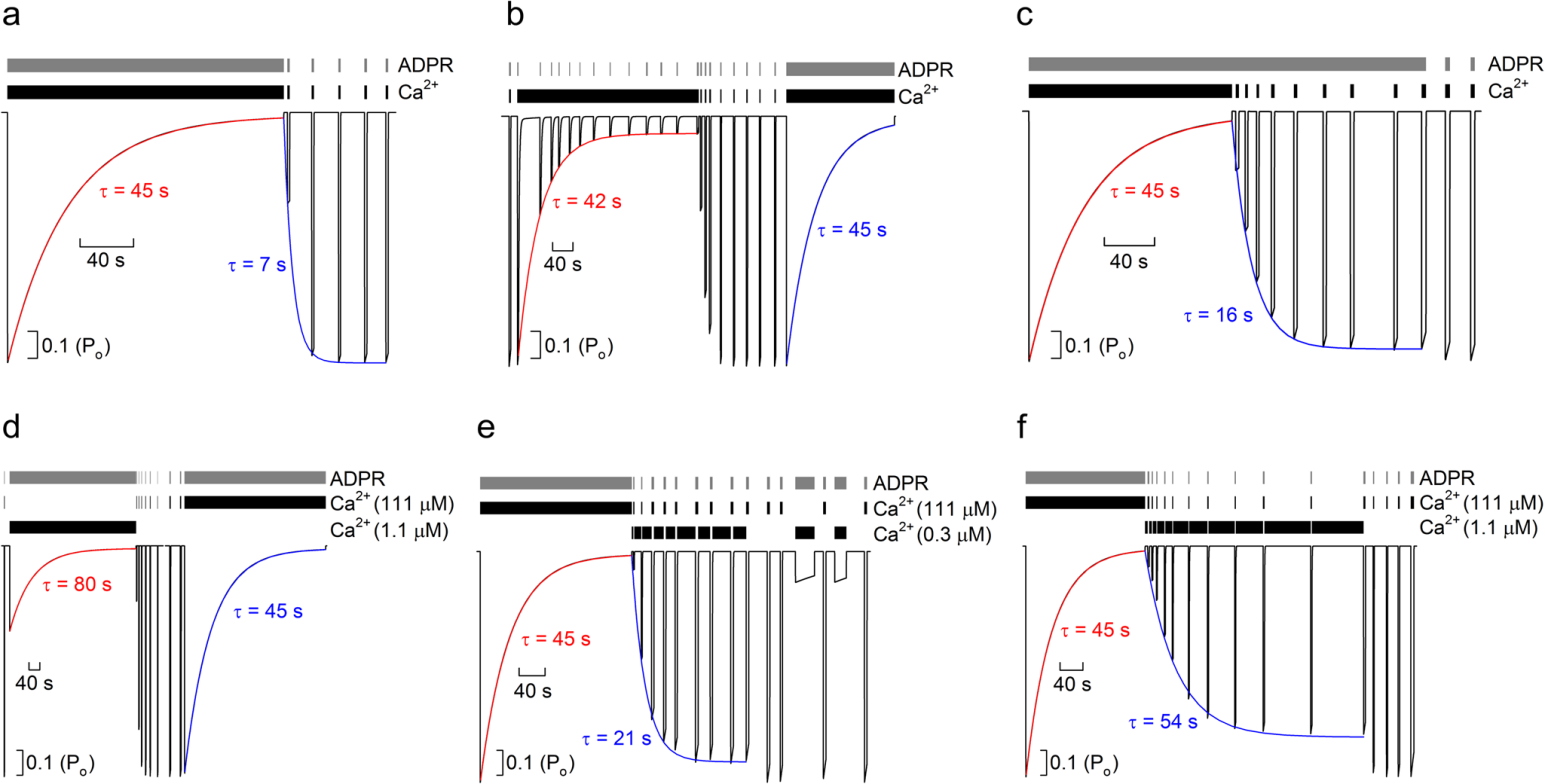
Open probability time courses predicted by the kinetic model recapitulate experimental current time courses. **a**–**f** Time courses of open probability (P_o_) calculated from the model in Fig. 3b for various experimental protocols. For each panel (**a**–**f**) the experimental protocol, depicted by the bars that represent agonist exposure (*black*, Ca^2+^; *gray*, ADPR), is exactly identical to that of the corresponding panel in Fig. 2. For the periods between *black bars* free [Ca^2+^] was set to 1 nM. Time courses of inactivation in (**a**, **c**–**f**), and of the second inactivation in (**b**), were fitted by single exponentials (*red* and *blue curves*, time constants plotted). For the time courses of recovery in (**a**, **c**, **e**, **f**) and of the first inactivation in (**b**) envelope curves (*red* and *blue curves*, time constants plotted) were obtained by fitting single exponentials to the maximum points of the current responses to the brief agonist exposures.

To validate the simple model with its optimized parameters (K_eq_ values printed in Fig. 3b, absolute values of inactivation and recovery rates in Fig. 3c), we examined how well it accounts for our experimental observations. For each of the six experimental protocols shown in Fig. 2a-f the predicted open probability time course (Fig. 4a-f), calculated from the model using standard Q-matrix methods^30^, was remarkably similar to the experimentally observed current time course (compare Fig. 4a-f to Fig. 2a-f). In particular, for all tested conditions, the time constants of inactivation and recovery predicted by the model (Fig. 3a, *gray bars*) were in good agreement with the experimentally observed time constants (Fig. 3a, *red bars*). Similarly, the predicted dose response curves for macroscopic current activation by Ca^2+^ and ADPR (Supplementary Fig. 2a-b, *gray symbols*) recapitulated the experimental curves (Supplementary Fig. 2a-b, *red symbols*). Thus, despite its simplicity, the model provides important mechanistic insight into drTRPM2 inactivation and recovery, and quantitates the strength of allosteric coupling between the activation gate, the inactivation gate, and the Ca^2+^ binding site. In particular, it predicts that closure of the inactivation gate increases the equilibrium constant for activation gate opening by ~4-fold, and the affinity for Ca^2+^ binding by ~700-fold.

### Kinetic model explains paradoxical experimental observations

Given the assumption that ADPR occupancy does not directly affect inactivation/recovery rates (Fig. 3b, *red edges*), two observed features of drTRPM2 inactivation/recovery might seem paradoxical at first sight, but are fully explained by the model.

First, why is the observed recovery time constant in 1 nM Ca^2+^ + 32 μM ADPR (τ = 12±1 s (n = 18), Fig. 3a, *right*, *1st red bar*; cf., Fig. 2c, *right*) more than twice as long as that in 1 nM Ca^2+^ + 0 ADPR (τ = 5.1±0.6 s (n = 19), Fig. 3a, *right*, *2nd red bar*; cf., Fig. 2a, *right*)? In the absence of ADPR, inactivated channels are distributed between states CI, CI^Ca^, and OI^Ca^ (Fig. 3a, *right cube*) and, when exposed to 1 nM Ca^2+^ alone, spend the majority (99.3%) of time in state CI. Thus, under such conditions the observed recovery rate approximates the rate constant for step CI → CA ( ~ 0.19 s^-1^, Fig. 3c, *right*, *bottom*). In contrast, in the presence of saturating ADPR, inactivated channels are distributed between states ${{\rm{CI}}}^{{{\rm{Ca}}}}_{{\rm{ADPR}}}$, and ${{\rm{OI}}}^{{{\rm{Ca}}}}_{{\rm{ADPR}}}$ (Fig. 3a, *left cube*). Because the equilibrium between the latter two states is strongly shifted towards ${{\rm{OI}}}^{{{\rm{Ca}}}}_{{\rm{ADPR}}}$ (K_eq_=218), even in the presence of just 1 nM Ca^2+^ the channels spend only 41.8% of time in the recovery-permissive state CI_ADPR_ (but 0.27% in ${{\rm{CI}}}^{{{\rm{Ca}}}}_{{\rm{ADPR}}}$ and 58% in ${{\rm{OI}}}^{{{\rm{Ca}}}}_{{\rm{ADPR}}}$), resulting in ~2.5-fold slower observable recovery rate.

Second, when exposed to 1.1 μM Ca^2+^, why do channels inactivate in the presence (Fig. 2d), but recover in the absence (Fig. 2f, *right*) of ADPR? In the presence of saturating ADPR + 1.1 μM Ca^2+^ the pool of active channels (Fig. 3b, *left cube*, *left vertical face*) is mostly found in states ${{\rm{OA}}}^{{{\rm{Ca}}}}_{{\rm{ADPR}}}$ (probability=37.1%) and CA_ADPR_ (probability=62.2%), and thus inactivates at an apparent rate (~0.012 s^-1^; Fig. 2d, *left*) which is a proportionately weighted average of the two respective inactivation rate constants (0.022 s^-1^ and 0.0064 s^-1^, Fig. 3c, *left*, *top* and *bottom*). At the same time, the pool of inactivated channels – distributed between states CI_ADPR_, ${{\rm{Ci}}}^{{{\rm{Ca}}}}_{{\rm{ADPR}}}$, and ${{\rm{OI}}}^{{{\rm{Ca}}}}_{{\rm{ADPR}}}$ – is essentially trapped in state ${{\rm{OI}}}^{{{\rm{Ca}}}}_{{\rm{ADPR}}}$, spending a negligible fraction (0.06%) of time in the recovery-permissive state CI_ADPR_. In contrast, in 0 ADPR + 1.1 μM Ca^2+^ the pool of active channels (Fig. 3b, *right cube*, *left vertical face*) accumulates in state CA (probability=98.9%) from which inactivation is slow (apparent inactivation rate ~0.0067 s^-1^), whereas inactivated channels – distributed between states CI, CI^Ca^, and OI^Ca^ – spend a non-negligible fraction (11.1%) of time in the recovery-permissive state CI (apparent recovery rate ~0.023 s^-1^). The balance between those apparent rates results in slow partial recovery (Fig. 2f).

### Inactivation/recovery mechanism is similar for hsTRPM2, but Ca^2+^ binding to inactivated channels is even tighter

**Fig. 5:**
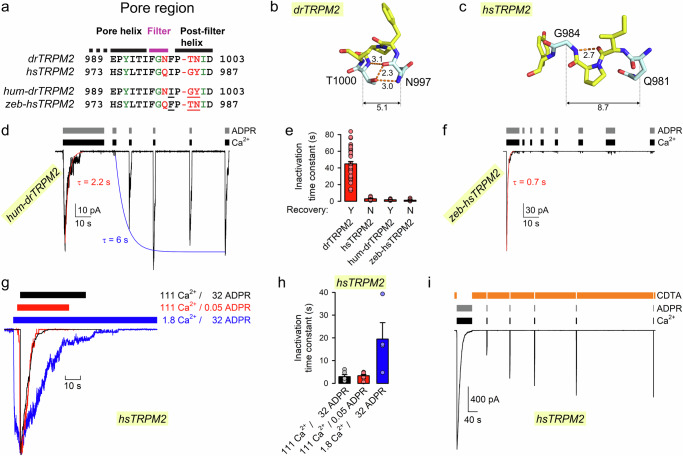
Pore sequence changes explain acceleration, and tighter Ca^2+^ binding irreversibility, of hsTRPM2 inactivation. **a** Alignment of pore sequences for hsTRPM2, drTRPM2, hum-drTRPM2, and zeb-hsTRPM2 channels. The three positions mutated in hum-drTRPM2 and zeb-hsTRPM2 are underlined. **b**, **c** Stick representation of the structures of (**b**) segment N997-N1001 of drTRPM2 (from PDBID: 6drk), and (**c**) corresponding segment Q981-Y985 of hsTRPM2 (from PDBID: 6mix). Residues N997 and T1000 in drTRPM2, and corresponding residues Q981 and G984 in hsTRPM2, are highlighted in *pale cyan*, with α-carbon distances marked. *Orange dashes*, hydrogen bonds. **d** Inactivation in 111 μM Ca^2+^ + 32 μM ADPR, and recovery in the absence of ligands, for hum-drTRPM2 channels. Experimental conditions as in Fig. 2a. **e** Time constants of inactivation of drTRPM2, hsTRPM2, hum-drTRPM2, and zeb-hsTRPM2 at 37 ^o^C, in the presence of 111 μM Ca^2+^ and 32 μM ADPR. Data for hsTRPM2 and hum-drTRPM2 are replotted from^28^. Bars represent mean ± S.E.M. from n independent patches, n = 39, 7, 9, and 12. **f** Inactivation in 111 μM Ca^2+^ + 32 μM ADPR, and lack of recovery in the absence of ligands, for zeb-hsTRPM2 channels. Experimental conditions as in Fig. 2a. **g** Time courses of activation and inactivation of hsTRPM2 in response to agonist exposure (*bars*) at 37^o^C, in the presence of 111 μM Ca^2+^ + 32 μM ADPR (*black trace* and *bar*), 111 μM Ca^2+^ + 0.05 μM ADPR (*red trace* and *bar*), and 1.8 μM Ca^2+^ + 32 μM ADPR (*blue trace* and *bar*). Experimental conditions as in Fig. 1a. **h** Time constants of inactivation of hsTRPM2 at 37^o^C, in the presence of the indicated combinations of agonist concentrations. Bars represent mean ± S.E.M. from n independent patches, n = (from left to right) 7, 8, and 4. **i** Inactivation of hsTRPM2 in the presence of 111 μM Ca^2+^ + 32 μM ADPR, and partial recovery in the presence of 0 ADPR + 10 mM CDTA (*orange bars*; free [Ca^2+^] low picomolar).

Following the abrupt sequence change in the post-filter region that happened between invertebrate and vertebrate TRPM2 orthologs (cf., Fig. 1c, *orange line*) that sequence continued to evolve in vertebrates, resulting in three further substitutions in hsTRPM2 relative to drTRPM2 (Fig. 5a). In cryo-EM structures of drTRPM2^20^ segment N997-T1000 adopts a compact helical arrangement stabilized, among others, by two hydrogen bonds between the threonine side chain and the asparagine backbone (Fig. 5b, *pale cyan sticks*). In hsTRPM2 cryo-EM structures^21^ the analogous segment is more extended, with less stabilizing interactions (Fig. 5c). In an earlier study^28^ “humanizing” the three evolving positions in drTRPM2 (Fig. 5a, “hum-drTRPM2”) was shown to accelerate inactivation by ~25-fold, rendering its rate comparable to that of hsTRPM2 (Fig. 5d; Fig. 5e, *first three bars*). Conversely, restoration of the three zebrafish residues into the hsTRPM2 pore (Fig. 5a, “zeb-hsTRPM2”) did not slow inactivation (Fig. 5f; Fig. 5e, *last bar*), suggesting that the stabilizing interactions present in drTRPM2 cannot be recovered in hsTRPM2 by simple reintroduction of the three zebrafish residues, as the corresponding positions are too far apart to interact (Fig. 5c, *pale cyan sticks*).

Interestingly, we found here that hum-drTRPM2 channels still fully recover upon ligand removal (Fig. 5d), in stark contrast to hsTRPM2 (Fig. 1a). In line with that observation, zeb-hsTRPM2 channels failed to recover (Fig. 5f), in stark contrast to drTRPM2 (Fig. 2a). Thus, the three additional post-filter substitutions do not explain lack of recovery of human TRPM2, raising the question whether the molecular mechanism of inactivation is indeed conserved between the two orthologs.

To further probe the mechanism of inactivation of hsTRPM2, we tested (Fig. 5g-h) how its rate is affected by lowering the concentrations of either agonist to roughly half of its EC_50_ value (cf. ^9^,). Compared to the presence of saturating concentrations of both agonists, lowering [ADPR] to 0.05 μM did not significantly (p = 0.69) affect the inactivation time constant (Fig. 5g, *black* vs. *red trace*; Fig. 5h, *black* vs. *red bar*). In contrast, lowering free [Ca^2+^] to 1.8 μM significantly (p = 0.013) slowed inactivation, prolonging its time constant by ~7-fold (Fig. 5g, *blue trace*; Fig. 5h, *blue bar*). The fact that lowering the concentration of one or the other agonist similarly reduces open probability but differentially affects inactivation rate implies that activation gate closure per se does not slow inactivation, contrary to earlier suggestions^19^.

All the above features of hsTRPM2 inactivation resemble those described here for drTRPM2 (Figs. 2b, 2d). Together with its reported temperature dependence in both orthologs (Supplementary Fig. 1c-d^28^), these data suggest that the mechanism of inactivation is conserved between the two species. Why then does hsTRPM2 not recover upon Ca^2+^ removal (Fig. 1a)? For drTRPM2 we have shown that inactivated channels bind Ca^2+^ more tightly. We therefore hypothesized that Ca^2+^ binding to the inactivated channel has evolved to be even stronger in hsTRPM2, such that lowering free [Ca^2+^] to ~1 nM is insufficient to unload Ca^2+^ from its binding site, leaving hsTRPM2 channels trapped in the Ca^2+^-bound inactivated state. To evaluate that possibility, we tested whether inactivated hsTRPM2 channels may be recovered by lowering free [Ca^2+^] to low picomolar, using 10 mM of the stronger Ca^2+^ chelator trans-1,2-Diaminocyclohexane-N,N,N′,N′-tetraacetic acid (CDTA) (Fig. 5i, *orange bars*). Indeed, superfusion of inactivated hsTRPM2 channels with a ligand-free solution containing 10 mM CDTA resulted in time-dependent recovery of the active channel pool, although recovery remained incomplete suggesting that the conformational equilibrium of the inactivation gate could not be fully shifted towards active. A more quantitative analysis of the [Ca^2+^] dependence of hsTRPM2 recovery will have to await determination of CDTA stability constants at 37 ^o^C, as well as the availability of Ca^2+^ probes sensitive in the picomolar range, to determine final free [Ca^2+^] in our bath solutions.

## Discussion

**Fig. 6:**
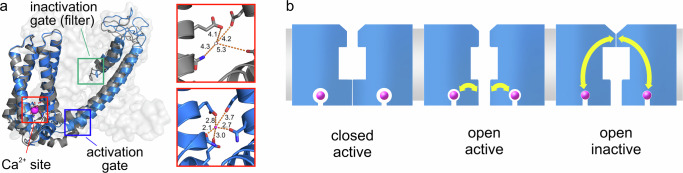
Allosteric coupling between the activation gate, the inactivation gate, and the Ca^2+^ binding site. **a** Structural alignment of the transmembrane domain (S1-S6) and the TRP helix of a subunit of drTRPM2, for the cryo-EM structures obtained in the ligand-free (PDBID: 6drk, *gray ribbon*) and in the ADPR+Ca^2+^-bound (PDBID: 6drj, *blue ribbon*) state. The structures were aligned through their S2-S3 segments. *Red*, *blue*, and *green boxes* highlight the positions of the Ca^2+^ site, the activation gate, and the inactivation gate (selectivity filter), respectively. The side chains of the residues involved in Ca^2+^ coordination, and of the conserved phenylalanine in the selectivity filter, are shown in stick representation. *Gray surface* represents the entire pore domain (S5-S6-TRP helix) of the tetramer. *Insets* show zoomed-in views of the Ca^2+^ site in the apo (*top*) and ligand-bound (*bottom*) structures, distances (in Å) between Ca^2+^ coordinating side chains and the Ca^2+^ ion (*bottom*, *purple sphere*) or its aligned position (*top*, *white sphere*) are indicated by *dashed lines*. **b** Cartoon representation of allosteric crosstalk (*yellow double-arrows*) between the Ca^2+^ site and the activation gate (*left* vs. *center*), and between the Ca^2+^ site and the inactivation gate (*center* vs. *right*). The three cartooned states correspond to states ${{\rm{CA}}}^{{{\rm{Ca}}}}_{{\rm{ADPR}}}$, ${{\rm{OA}}}^{{{\rm{Ca}}}}_{{\rm{ADPR}}}$, and ${{\rm{OI}}}^{{{\rm{Ca}}}}_{{\rm{ADPR}}}$ of the model in Fig. 3, respectively. Ca^2+^ binding becomes tighter upon opening of the activation gate, and even tighter upon closure of the inactivation gate.

Inactivation is an important feature of many neuronal ion channels, necessary for shaping their current responses to depolarization or agonists, and is typically a reversible process. The human TRPM2 channel is unique, in that its inactivation has been found irreversible^18^. Mutagenesis studies^19,23,28^ and the simultaneous evolutionary appearance of pore sequence changes and inactivation^26^ outlined the extracellular selectivity filter as the “inactivation gate” (Fig. 6a, *green box*), distinct from the cytosolic “activation gate” (Fig. 6a, *blue box*) formed by the four-helix bundle of the S6 C-termini^20^. On the other hand, information on the mechanism of inactivation, or on the reason for its irreversibility, was lacking.

Here we show that in the zebrafish, an early vertebrate, TRPM2 inactivation is fully reversible. That discovery allowed us to systematically study for the first time the mechanism of the process. We show here that inactivation and recovery are not directly influenced by ADPR binding, but strongly modulated by binding of Ca^2+^ (Fig. 2b, c). In principle, the Ca^2+^ binding site that modulates inactivation gating might be distinct from the “activating site” (Fig. 6a, *red box*) that regulates activation gating. However, multiple facts argue against such a possibility. An additional cytosolic Ca^2+^ site recently discovered in the close homolog TRPM4^31^ is not conserved in TRPM2. Regulation of inactivation and recovery by an interaction of permeating Ca^2+^ ions with the selectivity filter would predict modulation of both processes by extracellular Ca^2+^ and potentially by voltage, none of which was observed (Supplementary Fig. 1a-b). Moreover, the apparent affinities of Ca^2+^ for modulating the rates of both inactivation (Fig. 2d) and recovery (Fig. 2e, f) are similar to that for gating activation (Supplementary Fig. 2a), allowing a simple model with a single Ca^2+^ binding site (Fig. 3b) to fully account for all three processes. Altogether these arguments support the notion that it is Ca^2+^ occupancy of the same binding sites, located between the cytosolic ends of the S2 and S3 helices (Fig. 6a, *red box*), that modulates the conformational changes of both the activation and the inactivation gate. We demonstrate here strong allosteric coupling between those three spatially distinct protein regions (Fig. 6a, *red*, *blue*, and *green boxes*).

Strong coupling between the Ca^2+^ site and the activation gate (Fig. 6b, *center*, *yellow arrows*) has been documented by multiple earlier studies. From a functional perspective, Ca^2+^ binding increases the closed-open equilibrium constant of the activation gate. Thus, by the thermodynamic principle of detailed balance, upon gate opening the Ca^2+^ site must undergo a conformational change which results in tighter Ca^2+^ binding: a feature demonstrated earlier for hsTRPM2^18^. From a structural perspective, that allosteric crosstalk is revealed by an alignment of the cryo-EM structures of drTRPM2^20^ obtained in the ligand-free closed and the fully liganded open state (Fig. 6a, *gray* and *blue ribbons*, respectively). Comparison of the Ca^2+^ sites in the two structures reveals that, simultaneously with the opening of the activation gate, the conserved side chains which coordinate the Ca^2+^ ion move substantially closer to the central ion binding position (Fig. 6a, *insets*; Fig. 6b, *left* vs. *center*). (These tendencies are also observed in cryo-EM structures of hsTRPM2, with the caveats of lower resolution and uncertainty of whether the ADPR+Ca^2+^-bound structures represent a fully open state^21,22^.)

We provide here strong functional evidence for a similar allosteric coupling between the Ca^2+^ site and the inactivation gate in drTRPM2 (cf., Fig. 6b, *right*, *yellow arrows*). Because Ca^2+^ binding promotes inactivation whereas unbinding promotes recovery (Fig. 2b, c), inactivated channels must necessarily bind Ca^2+^ more tightly. Our quantitative model suggests that inactivation gate closure increases the equilibrium constant for Ca^2+^ binding by ~700-fold (Fig. 3b, compare two *blue edges* of *bottom faces*). Finally, the fact that inactivation becomes more complete for open than for closed channels (Fig. 2b, *left* vs. *right*) implies allosteric coupling between the activation and the inactivation gate: inactivation gate closure increases the equilibrium constant for activation gate opening (Fig. 3b, compare two *black edges* of *right vertical faces*). How, at a structural level, the conformational changes of the activation gate or Ca^2+^ site are transmitted to the spatially distant inactivation gate (selectivity filter) is unclear, but the cryo-EM structures do provide a hint. Comparison of the apo and fully liganded drTRPM2 structures reveals a clear change in distance between the selectivity filter and the Ca^2+^ site, indicative of a propagated, long-range conformational rearrangement (Fig. 6a, *green box*, compare positions of the phenylalanine side chains in the selectivity filter).

The simplified kinetic model developed here considers only a single Ca^2+^ and a single ADPR binding site (Fig. 3b), but nevertheless allows a remarkably accurate description of all the functional features observed in this study (see Fig. 2 vs. Fig. 4; *red* vs. *gray bars* in Fig. 3a; *red* vs. *gray symbols* in Supplementary Fig. 2). (For a true tetrameric channel with 4 Ca^2+^ and 4 ADPR binding sites, the vertices of the cubes depicted in Fig. 3b represent only channels with either 0 or 4 agonists bound to either type of binding site.) One feature that the simplified model does not predict is the slight sigmoidicity of the observed inactivation and recovery time courses, both of which typically start with a slight delay. (Due to the low time resolution of sampling, for recovery that delay is resolved only under conditions that sufficiently slow the process (e.g., Fig. 2f).) That sigmoidicity might be explained by concerted conformational changes of the inactivation gate that follow prior structural rearrangements in all four subunits of a homotetrameric channel. Nevertheless, the simple model developed here will be useful for the interpretation of future quantitative studies on TRPM2 inactivation/recovery.

We show here that – despite apparent phenotypic differences – the basic molecular mechanisms of inactivation and recovery are shared between drTRPM2 and hsTRPM2. For both orthologs the rate of inactivation is modulated by Ca^2+^ but not ADPR binding (Figs. 2b, d, 5g-h), and is modestly temperature sensitive (Supplementary Fig. 1c-d;^28^). Evolutionary changes in the structure of the post-filter region in hsTRPM2 (Fig. 5a-c) explain the much faster inactivation rate (Fig. 5d-e) but not the lack of recovery of the human ortholog (Fig. 5e). The latter unique feature reflects trapping of the human channel in an inactivated state which binds Ca^2+^ too tightly to allow its release at physiological resting free [Ca^2+^], requiring free [Ca^2+^] to be reduced to picomolar levels for recovery (Fig. 5i). Interestingly, for hsTRPM2 channels recovered by CDTA treatment inactivation in the presence of ADPR + Ca^2+^ appeared much accelerated, causing loss of a substantial fraction of the recovered pool within each brief (1-2 s) time window of ligand exposure and thus incomplete recovery (Fig. 5i). This suggests that CDTA treatment, in addition to unloading Ca^2+^ from the activating sites, may cause some further structural alteration. Possibly in the presence of CDTA other structurally important, but as yet unidentified, metal ions might be removed from the protein. Along those lines, structural studies on drTRPM2 identified a conserved N-terminal Zn^2+^ binding domain essential for structural integrity^32^. Additional cytosolic binding sites for Mg^2+^ and Ca^2+^ ions, respectively, were described for the TRPM2 ortholog from the choanoflagellate *Salpingoeca rosetta*^24^ and for human TRPM4^31^.

In conclusion, it appears that vertebrate evolution has tuned the inactivated state of TRPM2 in humans to require non-physiologically low free [Ca^2+^] for recovery. It thus seems unlikely that in a live human cell the TRPM2 channel on its own should be able to recover by unloading its bound Ca^2+^. From a physiological perspective, the irreversibility of hsTRPM2 inactivation raises several intriguing questions to be addressed by future studies. First, given the evidence for hsTRPM2 activation by a self-generated Ca^2+^ nanodomain^9,10^, rapid irreversible inactivation of hsTRPM2 might ensure that the resulting Ca^2+^ spark remains localized. E.g., it will be interesting to investigate whether in the POA, where TRPM2 is found in presynaptic boutons^11^, [Ca^2+^] signaling through TRPM2 remains limited to a single bouton. A second intriguing question is whether there may be other helper proteins in the cell that facilitate Ca^2+^ unbinding and thus channel recovery, or whether hsTRPM2 has been truly developed to function as an ephemeral “disposable” ion channel which is recycled after a single bout of activity. Interestingly, TRPV1 channels also irreversibly inactivate upon sustained exposure to noxious heat, and such single-use mode has been argued to offer a biological advantage that pays off for the investment of resynthesizing the channel pool^33^.

Under physiological conditions, the strong acceleration of hsTRPM2 inactivation rate by Ca^2+^ (Fig. 5g-h) provides a further unexpected link between hsTRPM2 response time courses and temperature. Within the limited temperature range experienced by hsTRPM2 channels in the brain and internal organs (37-40^o^C) the modest intrinsic temperature sensitivity of its inactivation rate (Q_10_ ~ 4) is unlikely to play a major role^28^. However, under physiological conditions local [Ca^2+^] around the activating sites of the channel was shown to become higher at 40^o^C compared to 37^o^C, due to the larger Ca^2+^ influx at the higher temperature^9^. Because in turn Ca^2+^ accelerates inactivation (Fig. 5g-h), in live cells the apparent Q_10_ of hsTRPM2 inactivation is likely larger than that observed at constant cytosolic [Ca^2+^]^28^, and should contribute to shaping the time courses of hsTRPM2 temperature responses.

For drTRPM2, which is not a “thermoTRP channel” as its gating itself is not temperature sensitive^28^, the large range of environmental temperatures (10-40^o^C) experienced by the zebrafish in their natural habitat^34^ suggests that even the modest temperature sensitivities of both inactivation^28^ and recovery rates (Supplementary Fig. 1c-d; Q_10_ ~ 3 and ~5, respectively) might become physiologically relevant for temperature sensing.

## Methods

### Molecular biology

The hum-drTRPM2/pcDNA3 and zeb-hsTRPM2/pcDNA3 constructs were generated from drTRPM2/pcDNA3 and hsTRPM2/pcDNA3, respectively, using the Quikchange II XL Kit (Agilent Technologies), and confirmed by automated sequencing (LGC Genomics).

### Expression of TRPM2 channels in HEK293 cells

HEK 293 T cells (ATCC, cat. # CRL-11268) transiently expressing drTRPM2 (or TRPM2 mutants) and green fluorescent protein (GFP) were generated by co-transfecting HEK 293 T cells with drTRPM2/pcDNA3 (or hum-drTRPM2/pcDNA3 or zeb-hsTRPM2/pcDNA3) and GFP/pcDNA3 at a 10:1 ratio (FuGENE HD transfection reagent, Promega). HEK 293 cells stably expressing hsTRPM2 were purchased from SB Drug Discovery. Cells were cultured at 37°C in 5% CO_2_, in DMEM medium supplemented with 4.5 g/L Glucose (Lonza), 10% FBS (EuroClone), 2 mM glutamate, and 100 units/ml penicillin/streptomycin (Lonza).

### Inside-out patch-clamp recordings

TRPM2 currents were recorded in inside-out patches from HEK 293 cells transiently expressing drTRPM2 or TRPM2 mutants, or stably expressing hsTRPM2, as described^9^. Pipette (extracellular) solution contained (in mM) 140 Na-gluconate, 2 Mg-gluconate_2_, 10 PIPES (pH 7.4 with NaOH), 1 EGTA (pH 7.4 with NaOH); free [Ca^2+^] was ~1 nM. To test the effects of extracellular Ca^2+^, EGTA was omitted from the bath solution resulting in free [Ca^2+^] ~ 0.3 μM (Supplementary Fig. 1b). Bath (cytosolic) solution contained (in mM) 140 Na-gluconate, 2 Mg-gluconate_2_, 10 PIPES (pH 7.1 with NaOH). Free [Ca^2+^] in the micromolar range was adjusted by titrating gluconate with Ca^2+^. For bath solutions with 1 nM - 1 μM free [Ca^2+^] 1 mM EGTA was added and titrated with Ca^2+^. To lower free [Ca^2+^] into the picomolar range, Mg^2+^ was omitted and EGTA replaced by 10 mM CDTA (Sigma-Aldrich, cat.# 32869). (Of note, omission of Mg^2+^ does not itself affect hsTRPM2 inactivation^19^.) For all solutions, except the one with CDTA, free [Ca^2+^] was spectrophotometrically determined both at 25^o^C and at 37^o^C^9^. For the CDTA-containing solution free [Ca^2+^] was estimated using MaxChelator and published stability constants for Ca^2+^ and Mg^2+^ determined at 25^o^C^35^. All bath solutions also contained 200 μM AMP to block endogenous TRPM4-like cation channels, and 10 μM dioctanoyl-PIP_2_ (Cayman Chemical) to maintain membrane PIP_2_ levels. The pipette electrode was placed into a 140 mM NaCl-based solution carefully layered on top of the Na-gluconate-based pipette solution, and the bath electrode was placed into 3 M KCl connected to the bath solution through a KCl-agar bridge^18^. Following excision from the cell, the patch was placed into a flow chamber which allowed rapid exchange (time constant <100 ms) of the continuously flowing bath solution using electronic valves (ALA-VM8, ALA Scientific Instruments). Bath temperature was controlled (TC-10 Dagan Corporation) and continuously monitored (BAT-12, Physitemp)^9^. Ca^2+^ (Ca-gluconate_2_; Sigma-Aldrich), ADPR (Sigma-Aldrich), dioctanoyl-PIP_2_, and AMP (Sigma-Aldrich) were dissolved into the bath solution from >100x concentrated, pH-adjusted aqueous stocks. Currents were recorded at a bandwidth of 2 kHz (Axopatch 200B; Molecular Devices), digitized at 10 kHz (Digidata 1322 A; Molecular Devices), and saved to disk (Pclamp10; Molecular Devices).

### Analysis of electrophysiological data

Inactivation time constants in the presence of fixed concentrations of ADPR + Ca^2+^ (Fig. 2a, b (*right*), c, d, e, f) were estimated from single-exponential fits to the current decay time courses. Inactivation time constant in the presence of 111 μM Ca^2+^ alone (Fig. 2b, *left*) was estimated by a single-exponential fit to the maximum points of the current responses to periodic brief exposures to 32 μM ADPR.

The kinetics of recovery under various conditions was roughly visualized by fitting single exponential “envelope curves” to the maximum points of the current responses evoked by periodic brief exposures to 111 μM Ca^2+^ + 32 μM ADPR (Fig. 2a, c, e, f; *blue curves*). Precise recovery time constants (Fig. 3a, *right*) were obtained by plotting the same maximum points as a function of cumulative time under the recovery condition (excluding the time intervals of brief agonist exposure), followed by single exponential fitting.

To obtain ligand dose response curves (Supplementary Fig. 2, *red symbols*), macroscopic fractional currents were calculated as the mean of the steady current under a test concentration of a ligand (ADPR or Ca^2+^) normalized to the mean of the steady-state currents in bracketing segments of record in the presence of a saturating ligand concentration recorded from the same patch. Dose response curves were fitted to the Hill equation using least squares.

### Determination of model parameters, and calculation of predicted open probability time courses

The kinetic parameters of the model in Fig. 3b were estimated as explained in the main text. Inactivation and recovery rates were directly estimated from the observed inactivation and recovery time constants. Compared to the rates of inactivation/recovery, activation gating and ligand occupancies are rapid equilibria for which only equilibrium constants could be confidently estimated from the ligand EC_50_ values and the observed open probabilities under liganded and unliganded conditions (see main text). Thus, for Ca^2+^ occupancy absolute rates (*k*_on_, *k*_off_) were assigned by assuming diffusion-limited binding (*k*_on_ = 6·10^9 ^M^-1^s^-1^). For ADPR occupancy absolute rates were assigned by assuming *k*_off_ ~ 0.5 s^-1^ from the open channel, to account for the ~2 s decay time constant following ADPR removal (cf., Fig. 2b, *left*). For activation gating absolute rates were assigned by assuming an opening rate of ~50 s^-1^ for the fully liganded channel, to account for the ~20 ms macroscopic relaxation time constant upon sudden addition of 111 μM Ca^2+^ + 32 μM ADPR.

Given the model, standard Q-matrix methods^30^ were used to calculate the predicted time courses of channel open probability for all six experimental protocols in Fig. 2a–f. The resulting predicted time courses (Fig. 4a–f) were then subjected to the same analysis described above for the experimental time courses, to obtain predicted inactivation/recovery time constants (Fig. 3a, *gray bars*). Open probability time courses for ligand titration experiments (cf. ^28^,) were similarly calculated from the model, and analyzed as described for the experimental time courses to obtain predicted ligand dose response curves (Supplementary Fig. 2, *gray symbols*).

### Statistics and reproducibility

All data are displayed as mean ± S.E.M., with the numbers (n) of independent biological replicates (patches) indicated in the figure legends; individual data values are also depicted (small circles). Statistical significances were evaluated using Student’s two-tailed t-test, differences were considered as significant for *P* < 0.05.

### Reporting summary

Further information on research design is available in the Nature Portfolio Reporting Summary linked to this article.

## Supplementary information

Supplementary Information

Supplementary Data

Description of Additional Supplementary Files

Reporting Summary

Transparent Peer Review file

## Data Availability

All data generated in this study are included in the main text and the figures, and the Supplementary Information. Source data for the graphs is available in the Supplementary Data file.
