# Supplementary material for: Ca2+ trapping by allosteric coupling explains species differences in TRPM2 inactivation reversibility
Source: Commun Biol. 2026 May 7;9:951. doi: 10.1038/s42003-026-10197-w (PMC13365451; doi:10.1038/s42003-026-10197-w)
Supplement: Supplementary file 1 — Supplementary Information [file 42003_2026_10197_MOESM1_ESM.pdf]

Supplementary Information

for

Ca^{2+} trapping by allosteric coupling explains species differences in TRPM2 inactivation reversibility

Adam Bartok[1,2,3] and László Csanády[1,2,3,*]

[1] Department of Biochemistry, Semmelweis University, Budapest, Hungary

[2] HUN-REN-SE Ion Channel Research Group, Budapest, Hungary

[3] HCEMM-SE Molecular Channelopathies Research Group, Budapest, Hungary

*Corresponding author:

László Csanády, M.D., Ph.D.
Semmelweis University
Department of Biochemistry
Tuzolto u. 37-47, Budapest, H-1094, Hungary
E-mail: csanady.laszlo@semmelweis.hu
Tel: (+36)-1-459-1500/60048
Fax: (+36)-1-267-0031

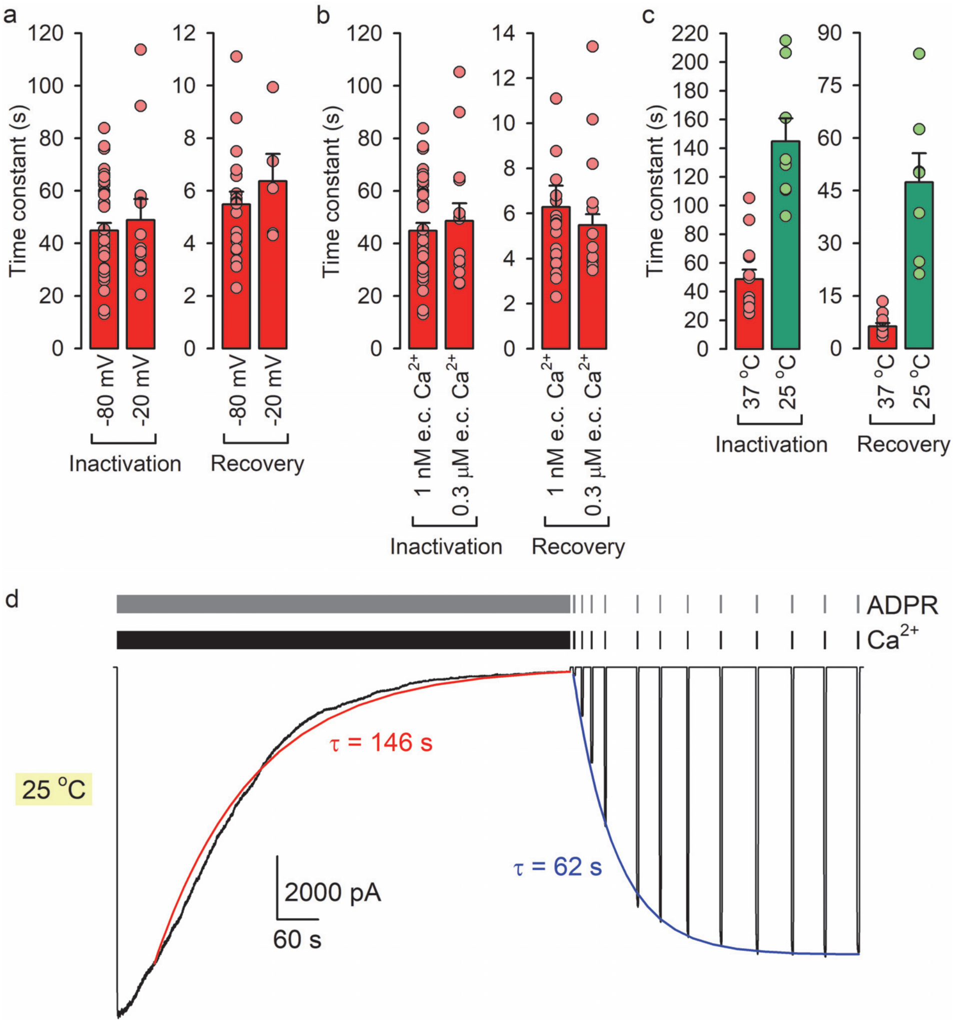

Supplementary Fig. 1. Dependence of drTRPM2 inactivation/recovery on membrane potential, extracellular Ca^{2+}, and temperature. (*a*) Time constants of drTRPM2 inactivation (*left*, in the presence of cytosolic 111 µM Ca^{2+} + 32 µM ADPR) and recovery (*right*, in the presence of cytosolic 1 nM Ca^{2+} + 0 µM ADPR) at the indicated membrane potentials. Temperature was 37°C, extracellular (pipette) [Ca^{2+}] was 1 nM. Bars represent mean±S.E.M. from n independent patches, n = (from left to right) 39, 12, 19, and 5. (*b*) Time constants of drTRPM2 inactivation (*left*, in the presence of cytosolic 111 µM Ca^{2+} + 32 µM ADPR) and recovery (*right*, in the presence of cytosolic 1 nM Ca^{2+} + 0 µM ADPR) in the presence of indicated concentrations of extracellular (pipette) [Ca^{2+}]. Temperature was 37°C, membrane potential was -80 mV. Bars represent mean±S.E.M. from n independent patches, n = (from left to right) 39, 14, 19, and 11. (*c*) Time constants of drTRPM2 inactivation (*left*, in the presence of cytosolic 111-133 µM Ca^{2+} + 32 µM ADPR) and recovery (*right*, in the presence of cytosolic 1 nM Ca^{2+} + 0 µM ADPR) at 37°C (*red bars*) and 25°C (*green bars*). Extracellular (pipette) [Ca^{2+}] was 0.3 µM, membrane potential was -80 mV. Bars represent mean±S.E.M. from n independent patches, n = (from left to right) 14, 8, 11, and 7. (*d*) Inside-out patch current from HEK-293T cell transiently expressing drTRPM2 evoked by cytosolic exposure to Ca^{2+} (133 µM, *black bars*) and ADPR (32 µM, *gray bars*). Membrane potential was -80 mV, temperature was 25°C, extracellular (pipette) [Ca^{2+}] was 0.3 µM. The time course of inactivation was fitted by a single exponential (*red curve*, time constant plotted). For the time course of recovery the envelope curve (*blue curve*, time constant plotted) was obtained by fitting a single exponential to the maximum points of the current responses to the brief agonist exposures.

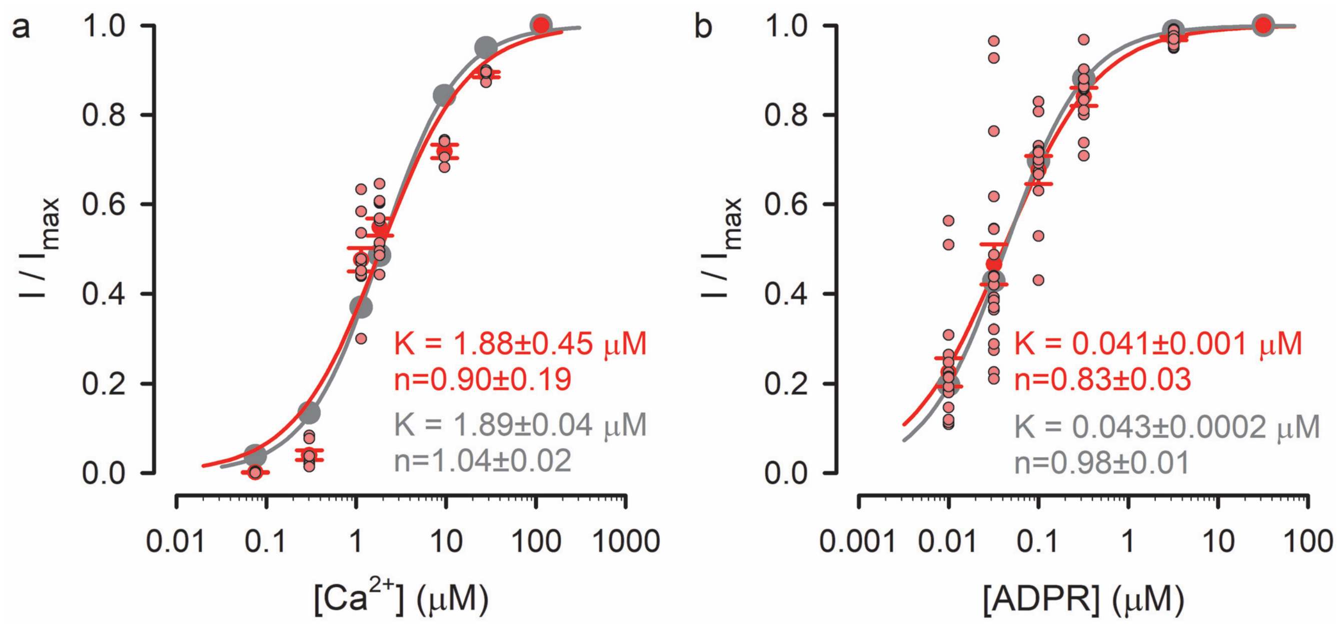

Supplementary Fig. 2. Dose response curves of drTRPM2 activation by agonists at 37°C. (*a*-*b*) *Red symbols* (replotted from[28]) represent fractional macroscopic currents of drTRPM2 in the presence of test concentrations of either Ca^{2+} (*a*) or ADPR (*b*), normalized to the average of the currents in bracketing segments of record in the presence of a saturating concentration of the agonist in the same patch. For the data in (*a*) [ADPR] was kept at 32 μM, for the data in (*b*) [Ca^{2+}] was kept at 111 μM. *Red symbols* represent mean±S.E.M. from n independent patches, n = 4-11 in (*a*), and 12-21 in (*b*). *Gray symbols* represent the calculated values of each data point, predicted by the model in Fig. 3b. *Solid lines* are fits to the Hill equation (*color coded*), with parameters plotted in the panels.
