# Supplementary material for: Ca2+ trapping by allosteric coupling explains species differences in TRPM2 inactivation reversibility
Source: Commun Biol. 2026 May 7;9:951. doi: 10.1038/s42003-026-10197-w (PMC13365451; doi:10.1038/s42003-026-10197-w)
Supplement: Supplementary file 5 — Transparent Peer Review file [file 42003_2026_10197_MOESM5_ESM.pdf]

nature portfolio

Peer Review File

Ca2+ trapping by allosteric coupling explains species differences in TRPM2 inactivation reversibility

Corresponding Author: Professor László Csanády

This manuscript has been previously reviewed at another journal. This document only contains information relating to versions considered at Communications Biology.

This file contains all reviewer reports in order by version, followed by all author rebuttals in order by version.

Version 0:

Reviewer comments:

Reviewer #1

(Remarks to the Author)
The authors have adequately responded to my comments and modified the manuscript accordingly. I have no further suggestions.

Reviewer #2

(Remarks to the Author)
The authors properly responded to the concerns raised by both reviewers and explained well the questions I asked. Now, I don't have any further concerns.

Open Access This Peer Review File is licensed under a Creative Commons Attribution 4.0 International License, which permits use, sharing, adaptation, distribution and reproduction in any medium or format, as long as you give appropriate

credit to the original author(s) and the source, provide a link to the Creative Commons license, and indicate if changes were made.
In cases where reviewers are anonymous, credit should be given to 'Anonymous Referee' and the source.
The images or other third party material in this Peer Review File are included in the article’s Creative Commons license, unless indicated otherwise in a credit line to the material. If material is not included in the article’s Creative Commons license and your intended use is not permitted by statutory regulation or exceeds the permitted use, you will need to obtain permission directly from the copyright holder.
To view a copy of this license, visit https://creativecommons.org/licenses/by/4.0/
